# Characterization of the Human Blood Virome in Iranian Multiple Transfused Patients

**DOI:** 10.3390/v15071425

**Published:** 2023-06-23

**Authors:** Marijn Thijssen, Gholamreza Khamisipour, Mohammad Maleki, Timothy Devos, Guangdi Li, Marc Van Ranst, Jelle Matthijnssens, Mahmoud Reza Pourkarim

**Affiliations:** 1 Laboratory for Clinical and Epidemiological Virology, Department of Microbiology, Immunology and Transplantation, Rega Institute for Medical Research, KU Leuven, 3000 Leuven, Belgium; marijn.thijssen@kuleuven.be (M.T.); marc.vanranst@kuleuven.be (M.V.R.); 2 Department of Hematology, Faculty of Allied Medicine, Bushehr University of Medical Sciences, Bushehr 75146-33196, Iran; ghr.khamisi@gmail.com; 3 Blood Transfusion Research Centre, High Institute for Research and Education in Transfusion Medicine, Tehran 14665-1157, Iran; malekim18@yahoo.com; 4 Laboratory of Molecular Immunology (Rega Institute), Department of Hematology, Department of Microbiology and Immunology, University Hospitals Leuven, KU Leuven, 3000 Leuven, Belgium; timothy.devos@uzleuven.be; 5 Hunan Provincial Key Laboratory of Clinical Epidemiology, Xiangya School of Public Health, Central South University, Changsha 410083, China; liguangdi.research@gmail.com; 6 Laboratory of Viral Metagenomics, Department of Microbiology, Immunology and Transplantation, Rega Institute for Medical Research, KU Leuven, 3000 Leuven, Belgium; jelle.matthijnssens@kuleuven.be; 7 Health Policy Research Centre, Institute of Health, Shiraz University of Medical Sciences, Shiraz 71348-14336, Iran

**Keywords:** microbiome, virome, blood transfusion, plasma, metagenomic sequencing, thalassemia, hemodialysis, healthy blood donor, anellovirus, Iran, Persian Gulf

## Abstract

Blood transfusion safety is an essential element of public health. Current blood screening strategies rely on targeted techniques that could miss unknown or unexpected pathogens. Recent studies have demonstrated the presence of a viral community (virobiota/virome) in the blood of healthy individuals. Here, we characterized the blood virome in patients frequently exposed to blood transfusion by using Illumina metagenomic sequencing. The virome of these patients was compared to viruses present in healthy blood donors. A total number of 155 beta-thalassemia, 149 hemodialysis, and 100 healthy blood donors were pooled with five samples per pool. Members of the *Anelloviridae* and *Flaviviridae* family were most frequently observed. Interestingly, samples of healthy blood donors harbored traces of potentially pathogenic viruses, including adeno-, rota-, and Merkel cell polyomavirus. Viruses of the *Anelloviridae* family were most abundant in the blood of hemodialysis patients and displayed a higher anellovirus richness. Pegiviruses (*Flaviviridae*) were only observed in patient populations. An overall trend of higher eukaryotic read abundance in both patient groups was observed. This might be associated with increased exposure through blood transfusion. Overall, the findings in this study demonstrated the presence of various viruses in the blood of Iranian multiple-transfused patients and healthy blood donors.

## 1. Introduction

Blood is an essential fluid of the human body, which is not only transfused to compensate loss through bleeding but also prescribed as a medicine [1]. Years after the establishment of modern blood banks and introduction of meticulous control on blood transfusion, blood safety is still a serious concern and an essential part of public health [2]. National and international health authorities strive to maintain a high level of blood transfusion safety by implementing appropriate strategies. For instance, strict guidelines in donor selection and the implementation of advanced pathogen detection techniques are applied to guarantee the safety of the blood transfusion practice [3,4,5].

The universal implementation of enzyme immunoassays and nucleic acid tests have significantly decreased the risk of transmitting bloodborne pathogens, including hepatitis b and c virus (HBV and HCV), human immunodeficiency virus (HIV), human T-cell lymphotropic virus (HTLV), and syphilis. Beyond the 68 transfusion-transmissible pathogens recognized by the American Association of Blood Banks [6], unknown or unexpected (bloodborne) pathogens and emerging infectious disease agents are still significant concerns for blood safety. The intensive contact with animals, through wildlife and domestic animal trade, viral vector expansion due to climate change, human mobility, and mass migration have allowed infectious agents to pass traditional endemic boundaries and disperse on a global scale [7,8,9]. In the last ten years, these demographic and global changes together with improved diagnostic assays have increased the detection of emerging and unexpected pathogens that can compromise blood transfusion safety [10,11]. 

The development of high-throughput sequencing technologies allowed a detailed assessment of human-derived and foreign genetic material in clinical and environmental samples in a non-targeted approach. Studies that applied these technologies and metagenomic analyses reported the presence of a viral community in the human blood, which was previously expected to be a sterile compartment in healthy conditions [12]. So far, little is known of the pathogenic potential, transmissibility, epidemiological pattern, and interaction with other segments of the human microbiome of these commensal-like viruses. Additionally, intriguing questions concerning the role of transfused blood as a potential carrier of these commensal-like viruses are still unanswered. These aspects raise concern over blood safety and vulnerability of frequently exposed individuals, such as immunocompromised and multi-transfused patients [13,14,15,16,17]. 

Certain health conditions require frequent blood transfusions, including hematological disorders and patients that need hemodialysis for the treatment of underlying kidney diseases. The frequent administration of blood products increases the risk of acquiring transfusion-transmitted infectious diseases in these patients. Even though the imposed safety measures have greatly reduced this risk, unknown pathogens and commensal-like viruses can still be transmitted. Studies have identified the presence of viral genomes in blood products, although they remain poorly characterized [18,19]. Hence, there is a demanding need to study these viruses in a blood transfusion context.

This study provides a detailed analysis of the blood virome in healthy blood donors and patients diagnosed with beta-thalassemia and patients that require blood transfusion to treat end-stage renal diseases (ESRD). The study population originates from the Boushehr Province, Iran, which lies in the “thalassemia belt”, a region that extends from the Mediterranean basin to south-eastern Asia. We will provide a detailed picture of the blood virome in this resource limited region by applying a metagenomic sequencing approach.

## 2. Materials and Methods

### 2.1. Study Population and Ethics Statement

This cross-sectional study was conducted from 2017 to 2018 in the Boushehr Province, Iran. Patients diagnosed with homozygous beta-thalassemia major (herein thalassemia patients) or ESRD patients receiving transfusion (herein hemodialysis patients) were included. These patients (multiple-transfused patients) receive red blood cell preparation on a frequent basis. The individuals were recruited in one of the five transfusion clinics located in Boushehr. In total, 155 thalassemia, 149 hemodialysis patients, and 100 healthy blood donors were included. Seven milliliters of blood were collected from each individual during blood transfusion or donation. Immediately after collection, plasma was separated from the samples and stored at −70 °C. Plasma samples were shipped under freezing conditions to the Laboratory for Clinical and Epidemiological Virology, Rega Institute, KU Leuven, Belgium. 

In addition to blood samples, demographic data including, sex, age, and the number of blood transfusions per month were received from the patient clinical record. This study was performed in accordance with the Declaration of Helsinki and approved by the ethical committee of the Bushehr University of Medical Sciences (IR.BPUMS.REC.1396.106). All participants gave written informed consent and were able to withdraw from the study at any moment. 

### 2.2. Plasma Virome Sequencing

Upon arrival, the samples were processed in the laboratory. Initially, the samples were centrifuged and 100 µL of the supernatant was pooled with five samples per pool. The pooled plasma samples were subjected to an adapted version of the NetoVIR protocol for viral particle enrichment and metagenomic sequencing [20]. To control for laboratory and environment contamination, negative controls were included in the different steps of the sample preparation procedure and pooled for sequencing. Pooled plasma samples and negative controls (H_2_O) were centrifuged for 3 min at 17,000× *g* and filtered through 0.8 µm polyether sulphone filters (Sartorius, Göttingen, Germany). To remove free-floating nucleic acids, the filtered samples were subjected to a nuclease treatment with a cocktail of 1 µL micrococcal nuclease (New England Biolabs, Ipswich, MA, USA) and 2 µL benzonase (Millipore, Burlington, United States) for 2 h at 37 °C. Both viral DNA and RNA were extracted (Viral RNA minikit, Qiagen, Hilden, Germany) and randomly amplified (including primary step of reverse transcription) with the Whole Transcriptome Amplification 2 kit (WTA2, Sigma Aldrich, Darmstadt, Germany) for 20 cycles. The amplification product was purified with the MSB SPIN PCRAPACE kit (Stratec, Birkenfeld, Germany) and prepared for sequencing by using the Nextera XT kit (Illumina, San Diego, CA, USA). DNA products were quantified with the Qubit fluorometer (Thermo Fisher Scientific, Waltham, MA, USA), and the High-Sensitivity DNA kit (Agilent, Ratingen, Germany) for the Bioanalyzer 2100 (Agilent, Ratingen, Germany) was used to determine the average library fragment size. Samples were pooled in equimolar ratios, and paired-end sequencing (2 × 150 bp) was performed on a NextSeq 500 Illumina platform (Nucleomics Core, Leuven, Belgium) with an average of 10 million reads per sample. 

### 2.3. Read Processing and Taxonomical Annotation

Following sequencing, Trimmomatic (v0.36) [21] was used to remove WTA2 and Nextera XT adapters and low-quality (parts of) reads. Furthermore, the 19 leading and tailing base pairs (bps) were removed. The trimming procedure was carried out with a sliding window of 4 base pairs with an average cut-off PHRED score of 20. Only reads with a minimal length of 50 bps were used for downstream analysis. Quality-controlled reads, which mapped against the human genome or a database of known contaminating sequences with Bowtie2 (v2.3.4.1), were removed [22]. The remaining reads were de novo assembled into contigs using metaSPAdes (v3.11.1) with kmer sizes of 21, 33, 55, and 77 bp [23]. Assembled contigs of sizes above 300 bps were clustered at 80% coverage and 95% nucleotide identity using nucmer from the MUMmer package (v3.23) [24,25]. Following clustering, quality-controlled reads were mapped back to the non-redundant contig database using bwa2 (v2.0), and a cut-off of 70% coverage defined the presence of a contig in a single sample [26]. The classification of contigs was carried out using ktClassifyBLAST on DIAMOND protein hits (database downloaded March 2021) and with BLASTn (E-value of 1 × 10^−10^) against the NCBI nt database (downloaded March 2021) [27,28,29]. For bacteriophage identification, VirSorter2 (v1.1.0) was used and CheckV (v0.8.1) to assess the completeness of viral genomes [30,31]. The mapped reads to the clustered contigs were normalized to allow comparison between pools. We calculated the reads per million kilobases (RPMK) and relative abundances of viruses in pooled samples. 

### 2.4. Phage Host Prediction

Host prediction of the identified phage contigs was done with two methods, (1) using clustered regularly interspaced short palindromic repeats (CRISPR) spacers, and (2) transfer-RNA (tRNA) sequences. CRISPR spacers were predicted from the bacterial RefSeq (NCBI) database (downloaded October 2020) using MinCED (v0.4.2) [32]. The predicted CRISPR spacers were subjected to a nucleotide BLAST search against the identified phage contigs (E-value of 1 × 10^−10^ and 100% identity). Host assignment was accepted with a minimum of two matches on the corresponding phage contig and common host. In addition to CRISPR spacers, tRNA genes were predicted from the phage sequences using Aragorn (v1.2.38) [33] and blasted on nucleotide level against bacterial sequences (E-value of 1 × 10^−10^, 100% identity).

### 2.5. Phylogenetic Analysis

From the assembled anellovirus contigs, intact open reading frame (ORF1) protein sequences were predicted with the “getorf” function of the EMBOSS software package [34]. MAFFT (v7.407) was used to align the identified ORF1 sequences with anellovirus reference sequences (*n* = 59, downloaded from NCBI Refseq, May 2022) using the iterative refinement method, and sequences were trimmed with trimAI (v1.1, gappyout setting) [35,36]. A phylogenetic tree was built under model LG+I+G4+F, determined by jModelTest (v2.1.10), with 1000 bootstrap replicates in RAxML (v8.2) [37,38]. A similar approach was adopted to build the pegivirus phylogenetic tree. Protein sequences of the non-structural protein 5b (NS5B, RNA-dependent RNA polymerase) were predicted using “getorf” and aligned with 28 reference sequences (downloaded from NCBI Refseq, July 2022) using MAFFT. The aligned sequences were manually trimmed, and RAxML with model LG+G4+F was applied to build the phylogenetic tree, with 1000 bootstrap replicates.

### 2.6. Quantitative PCR

A quantitative PCR (qPCR) method was adopted from available literature to evaluate the anellovirus quantity in the pooled samples [39]. The optimized qPCR protocol was developed to target the untranslated region with the following primers and probe: AMTS fwd (5′-GTGCCGNAGGTGAGTTTA-′3), AMTAS rev (5′-AGCCCGGCCAGTCC-′3), AMTASgr4 rev (5′-AGCCCGGCCAGACC-′3), and AMTPTU probe (5′-FAM-TCAAGGGGCAATTCGGGCT-BHQ1-′3. The samples were subjected to a qPCR program that started with a preheating step up to 95 °C for 10 min, followed by 95 °C for 10 s and 55.0 °C for 30 s for 40 cycles. 

### 2.7. Statistical Analysis

All statistical analyses were performed and visualized in R using vegan (v2.5-7), phyloseq (v1.34.0), bios2mds (v1.2.3), and ggplot2 (v3.3.5) packages [40,41,42,43,44]. Analysis of single variables in two groups was performed with the Wilcoxon rank-sum test. The Kruskal–Wallis test was used to compare multiple groups (e.g., read count, relative abundance, alpha diversity etc.), and a post-hoc Dunn’s test was used for pairwise comparison with the Benjamini–Hochberg correction (false-discovery rate (FDR) set at <0.05). 

## 3. Results

### 3.1. Population Characteristics

Blood samples from 155 beta-thalassemia, 149 hemodialysis and 100 healthy blood donors were selected for this study. Samples were pooled per five, resulting in 31, 32, and 20 pools, respectively. Thalassemia patients were relatively younger with a mean age of 24.7 + 8.5 years compared to 45 + 10.4 years in the hemodialysis group and 41 + 9.7 years in the blood donor population (Table 1, Kruskal–Wallis test with post-hoc Dunn’s test, *p* < 0.05). Males were more predominant in the thalassemia and control groups compared to hemodialysis patients. The number of transfused blood products per year/month were significantly higher in hemodialysis patients compared to thalassemia patients (Wilcoxon rank-sum test, *p* < 0.05). Both patient groups relied on the transfusion of red blood cells to treat the underlying disease.

### 3.2. Sequencing Data

**Figure 1:**
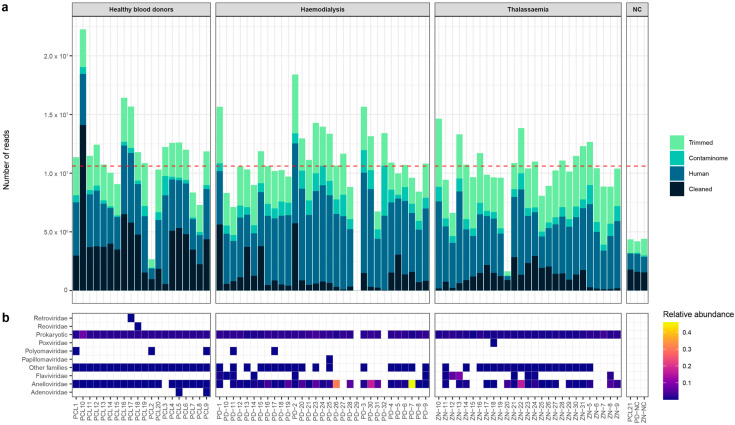
Processing of raw sequencing reads and relative abundance of frequently observed viral families per pool. (**a**) Fraction of the total number of generated reads that were removed during the processes of trimming and mapping to contamination sequences and human genome. The cleaned reads represent the fraction of total reads used for downstream analysis. Red dotted line: average number of reads calculated from all samples. (**b**) A heatmap of the relative abundance of the most frequently observed viral families and prokaryotic viruses per sequenced pool. NC: negative control.

The analyzed blood samples were sequenced with an average depth of ~10,600,000 reads (std ± 3,356,987, Figure 1a). Sequential steps of quality control, trimming, and host/contamination removal resulted in an average of 1,980,000 (std ± 2,100,000) reads for de novo assembly. In total, reads were assembled in contigs with a maximum length of 178,106 bps. Only contigs with a length above 300 bps were selected for downstream analysis. 

From the reads that mapped to selected contigs, an average of 131,350 (std ± 820,400) reads were classified as viral by DIAMOND and Blastn. To decrease the number of false positives, a minimum cut-off of 70% nucleotide coverage was used to assign the presence of a single contig. Furthermore, sequences detected in negative control samples were removed from the patient samples and excluded for further analysis. A variety of viral families were detected with different abundances in the blood samples (Figure 1b). Among the highly frequent families, viruses of the eukaryotic *Anelloviridae* family were detected in 65/78 (83%) of the pooled samples (Figure 1b). In addition to eukaryotic viruses, the majority of the putative viral contigs were classified as prokaryotic viruses by VirSorter2 and CheckV. Contigs were annotated based on amino acid and nucleotide homology searches. To assess the annotation quality, multiple parameters were evaluated including viral prevalence in the pooled samples, contig length, query coverage, and percentage amino acid identity with the closest hit in the reference database (Supplementary Figures S1–S4). Among the highly prevalent viral families, contigs of the *Anelloviridae* family were observed in the majority of analyzed pools. For most of the detected viral families, only small genome fragments were retrieved (Supplementary Figure S5) with a length below 500 bps. The longer fragments were observed for the *Anelloviridae* and *Flaviviridae* families as well as families of the *Caudovirales* order. For multiple sequences, the origin can be disputed and they might emerge from environmental or laboratory contamination. Therefore, contigs that were highly prevalent in the negative controls were removed and discarded from further analysis. Remarkably, a high number of the frequently detected prokaryotic viruses were observed in pools of healthy blood donors.

### 3.3. Eukaryotic and Prokaryotic Virome

The observed and annotated contigs can be divided in two major classes of viruses: (i) eukaryotic and (ii) prokaryotic viruses. The majority of viruses that belong to the eukaryotic virome are part of the *Anelloviridae* and *Flaviviridae* families (Figure 1b). Furthermore, viruses of the *Curciviridae*, *Circoviridae*, and *Parvoviridae* have been frequently observed (Supplementary Figures S1–S4). However, their wide distribution in the studied samples, in combination with low coverage and amino acid identity in the database, suggests that these families are potential contaminations and/or misclassified contigs, respectively. 

**Figure 2:**
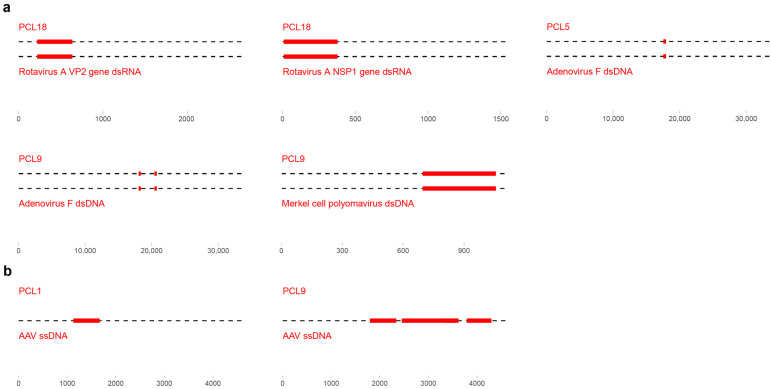
Genome segments detected of low-frequent viruses. (**a**) Schematic overview of the segments observed of double-stranded DNA or RNA viruses that were low-prevalent in the pooled sample. (**b**) Schematic overview of the detected genome segments of low-frequent single-stranded DNA viruses.

In addition to the prevalent families, some viruses were only observed in a few or single samples of healthy blood donors (PCL). The contigs only covered a limited area of the corresponding genomes, albeit with a high nucleotide identity of >95% to the closest hit in the database. In pool PCL18, two contigs were assembled and identified as part of the VP2 (inner capsid protein, 416 bps) and NSP1 (non-structural RNA-binding protein, 369 bps) gene of rotavirus A (Figure 2a). Pool PCL5 was found positive for adenovirus F (406 bps). Similarly, two fragments of adenovirus F were identified in pool PCL9 (321 and 349 bps) and an additional contig of Merkel cell polyomavirus (361 bps). Finally, contigs of the adeno-associated virus were assembled from pools PCL1 (*n* = 1542 bps) and PCL9 (*n* = 3544, 1080, and 508 bps) (Figure 2b). These findings indicate that in addition to the frequently observed viruses of the *Anelloviridae* and *Flaviviridae*, other viral families can be found in the blood of healthy individuals.

Multiple observed contigs were annotated as prokaryotic based on nucleotide and amino acid homology searches in the NCBI database. However, these databases are not well-curated for the detection and annotation of prokaryotic viruses. Therefore, we defined the presence of prokaryotic viruses based on the results of VirSorter2 (maximum score > 0.5) and CheckV (minimal completeness estimate of “low quality”). Combined with the annotation data from DIAMOND and Blastn, we found that on average 65 + 34% of viral reads were attributed to bacteriophages, while 35 + 34% of reads belonged to eukaryotic viruses. Host prediction of the observed prokaryotic contigs revealed *Escherichia*, *Cronobacter*, and *Citrobacter* genera as the hosts of prokaryotic viruses in samples of hemodialysis patients, while the Corynebacterium genus was the host in a single pool of healthy blood donors (Supplementary Figure S6). Host prediction was carried out based on presence of CRISPR spacers (supplementary Figure S6a) in the assembled genome and tRNA genes (Supplementary Figure S6b). tRNA genes (*n* = 15) were only observed for Corynebacterium phages.

**Figure 3:**
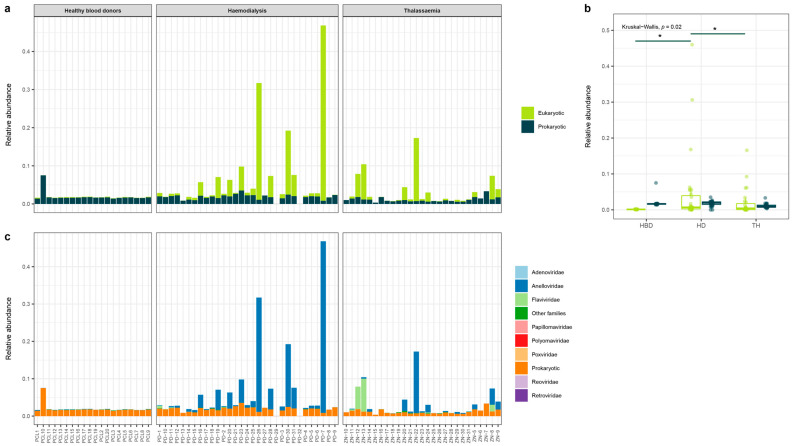
Eukaryotic and prokaryotic viruses observed in the blood of healthy blood donors and multiple transfused patients. (**a**) Relative abundance of eukaryotic and prokaryotic viruses per pool. (**b**) Boxplot of the eukaryotic and prokaryotic relative abundance per study group. Kruskal–Wallis test, *p* < 0.05, * post-hoc Dunn’s-test for pairwise comparison with Benjamini–Hochberg correction, *p* < 0.05. (**c**) Overview of the relative abundance of the individual eukaryotic viral families and prokaryotic viruses. HBD: healthy blood donors, HD: hemodialysis, TH: thalassemia.

Differences in sequencing depth can be a confounding factor in the comparison of the virome composition of individual samples. To compare the eukaryotic and prokaryotic load between the pooled samples, we normalized the data according to RPKM. The observed prokaryotic fraction of the blood virome remained relatively stable across the studied samples in terms of relative abundance of the total number of cleaned reads (Figure 3a,b). In contrast, the abundance of eukaryotic reads showed a high variability between different pools. Remarkably, the eukaryotic load was higher in samples of hemodialysis patients compared to healthy blood donors and thalassemia patients (Kruskal–Wallis test: *p* = 0.02) (Figure 3b). A more detailed analysis revealed that differences in eukaryotic viral abundance were mostly driven by the presence of the *Anelloviridae* and *Flaviviridae* (human pegivirus) viruses (Figure 3c, Supplementary Table S1). Two hemodialysis pools were negative for viral reads (PD-29 and PD-32), which could be associated with poor quality of the sequencing data in these samples. 

### 3.4. Anelloviridae Family

**Figure 4:**
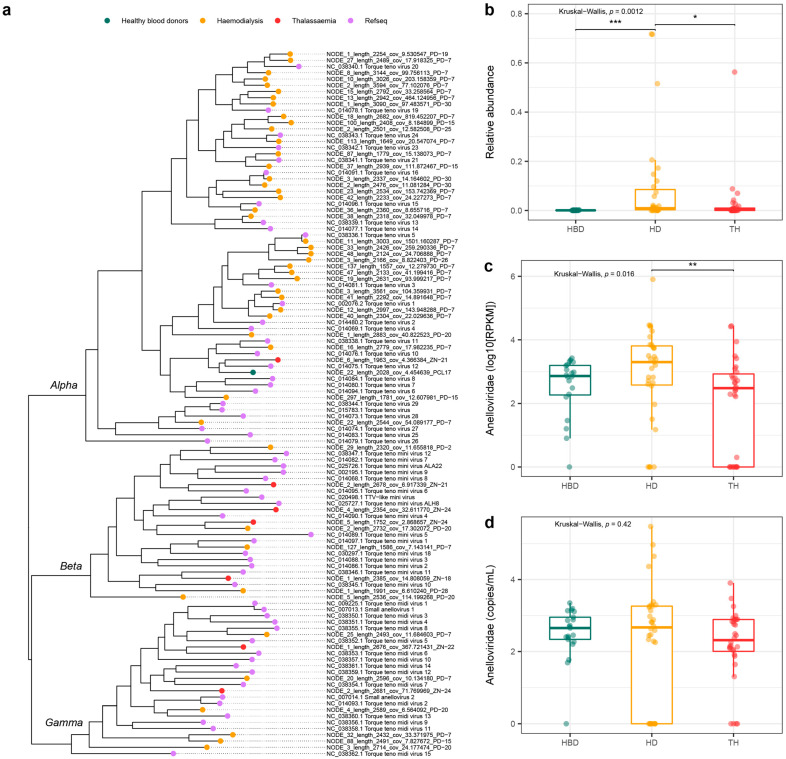
Phylogenetic analysis of the observed viruses of the *Anelloviridae* family and their abundance. (**a**) Maximum-likelihood tree of the predicted anellovirus ORF1 protein sequences from the pooled samples and NCBI reference sequences. (**b**) Relative abundance of the *Anelloviridae* family in the three study groups. (**c**) The absolute number of reads in RPKM among the study population. (**d**) Quantification results of the *Anelloviridae* family targeted by qPCR. Kruskal–Wallis test, *p* < 0.05, post-hoc Dunn’s test for pairwise comparison with Benjamini–Hochberg correction, *p* < 0.05. * *p* < 0.05. ** *p* < 0.01. *** *p* < 0.001. HBD: healthy blood donors, HD: hemodialysis, TH: thalassemia, RPKM: reads per kilobase of transcript per million reads mapped.

Among the widely abundant eukaryotic viral families, viruses of the *Anelloviridae* were most frequently observed in all three study groups (controls (PCL) 19/20, dialysis (PD) 27/31, and thalassemia (ZN) 19/27). Downstream annotation of the observed sequences identified the three established genera within the *Anelloviridae* family that are known to infect humans, the *Alphatorquevirus*, *Betatorquevirus*, and *Gammatorquevirus*. A total number of 720 anellovirus contigs were identified in the studied pools, with the highest proportion observed in dialysis pools (mean number of contigs per pool 25 ± 36). However, the number of contigs observed per pool were highly variable within this subgroup. The majority of contigs had a length <1 kb, while an increased abundance of fragments above 2 kb was found in the dialysis group (Supplementary Figure S7). Anellovirus classification is determined by the pairwise comparison of open reading frame 1 (ORF1) nucleotide sequences, with an average length of 1.6–1.8 kb. We were able to predict 54 ORF1 sequences from the observed contigs and build a phylogenetic tree with 59 reference sequences (Figure 4a, Supplementary Table S2). The tree confirmed the presence of all three genera known to infect humans across the studied samples. ORF1 sequences retrieved from dialysis pools were over-represented in the tree, followed by thalassemia pools, and a single complete ORF1 observed in control pools. Most patient ORF1 sequences clustered within *Alphatorque*- (*n* = 37), *Betatorque*- (*n* = 9), and *Gammatorquevirus* (*n* = 8) clades, respectively. 

#### 3.4.1. Number of Reads VS Abundance of Anellovirus

The relative abundance of the *Anelloviridae* members, corrected for sequencing depth, demonstrated differences in the three patient groups. The highest abundance was attributed to dialysis patients followed by thalassemia and control individuals (Figure 4b, Kruskal–Wallis test: *p* = 0.001). The absolute observed number of anellovirus reads were significantly increased in dialysis compared to thalassemia patients (Figure 4c, Kruskal–Wallis test: *p* = 0.02). In contrast, quantification of anelloviruses with qPCR showed no differences in anellovirus copies per mL between the three different groups (Figure 4d, Kruskal–Wallis test, *p*-value = 0.42). The qPCR method identified 72 (87%) positive pools for anelloviruses. The sequencing method was positive for 63 of the 72 qPCR positive pools, which results in an 88% sensitivity for the used sequencing method with qPCR as the reference method. Interestingly, eight pools were found negative in the qPCR method, while metagenomic sequencing and downstream analysis identified the presence of anellovirus contigs. 

#### 3.4.2. Anellovirus Diversity

**Figure 5:**
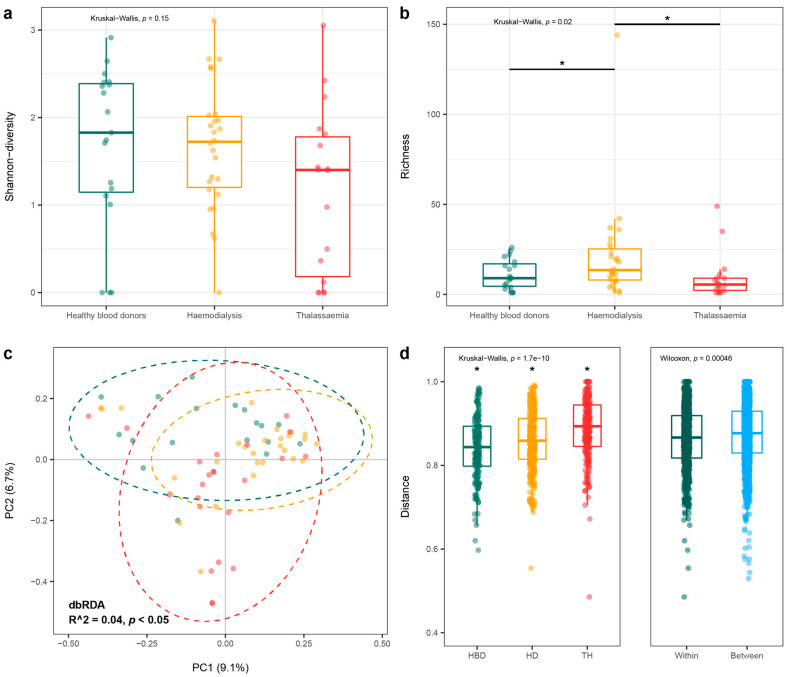
Diversity of the *Anelloviridae* family. (**a**) Shannon-diversity (within pool diversity) of the observed anellovirus contigs in individual pools compiled in the different study groups. (**b**) Richness of the anellovirus contigs in the pooled samples. (**c**) Principal components analysis on the UniFrac distance. UniFrac distance was calculated on the aligned ORF1 sequences predicted from the observed contigs or genomes that were extracted from the closest hit in the NCBI database. Dots are colored according to the study groups. dbRDA: distance-based redundancy analysis. (**d**) Distances between individual dots of the principal components analysis plots, according to study group (between, Kruskal–Wallis test with post hoc Dunn’s test (all *p* < 0.05), and within groups, Wilcoxon signed rank-test). HBD: healthy blood donors, HD: hemodialysis, TH: thalassemia, Within: within group, Between: between group. * *p* < 0.05.

Annotation of the assembled anellovirus contigs revealed the presence of various species. We have applied two different annotation strategies, which included a nucleotide and protein search against the NCBI database. Contigs with similar hits to the reference database (>90% nucleotide similarity with closest hit) were compiled and considered a single virus in the downstream analysis. With these settings, both alpha diversity (Shannon) and anellovirus richness were calculated for all individual pools and grouped into control, dialysis, and thalassemia conditions. Even though the alpha diversity was similar between the different groups (Kruskal–Wallis test: *p* = 0.15), anellovirus richness was increased (Kruskal–Wallis and post-hoc Dunn’s test *p* < 0.05 with FDR correction) in the dialysis patients (Figure 5a,b).

Besides the within-pool diversity, we performed an unweighted Unifrac distance analysis to define the between sample diversity, i.e., beta-diversity. This method accounts for phylogenetic relationship between sequences and presence and absence of specific viruses. Since only a limited number of intact ORF1 sequences were retrieved from the dataset, we predicted ORF1 from the closest hit in the reference database (a positive hit was considered with >90% nucleotide identity). A principal components analysis on the Unifrac distance did not result in the clustering of samples according to disease condition (Figure 5c). Even though disease condition seemed to significantly contribute to the spread of the individual samples, it only accounted for a small fraction of the total variation (dbRDA: R^2^ = 0.04, *p* < 0.05). The distances between individual points inside a patient group were the largest for thalassemia patients, while the lowest distance was observed for control patients (Figure 5d, Kruskal–Wallis test: *p* < 0.05). This suggests that the control pools have a less diverse anellovirus population. Overall, samples from the same population (healthy versus patient) are more closely related compared to samples between groups (Figure 5d, Wilcoxon signed-rank test, *p* < 0.05). However, these differences are very limited (within group, 0.86, and between group, 0.87).

#### 3.4.3. *Anelloviridae* Genera

**Figure 6:**
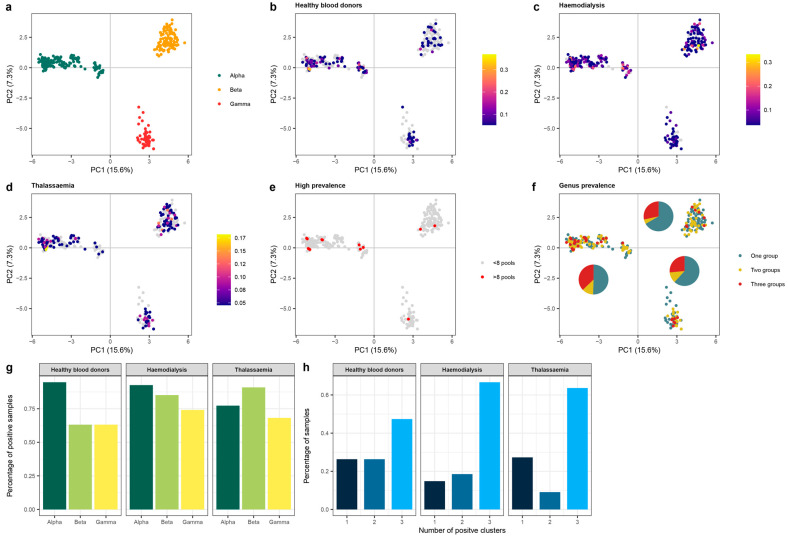
The evolutionary space of the observed members of the *Anelloviridae* family and genus distribution. (**a**) Principal components analysis on the aligned ORF1 anellovirus sequences predicted from the observed contigs or closest hit in the NCBI database. Dots are colored according to the anellovirus genus. (**b**–**d**) The similar principal components analysis as in panel A, albeit the dots are colored according to the percentage of positive pools within the corresponding study group. (**e**) Dots colored according to the prevalence in the total study population, red indicating the prevalence in more than 8 pools. (**f**) The prevalence of anellovirus sequences in one or more study groups. Pie charts indicate the proportion of contigs that were shared by one or more study groups per cluster (i.e., anellovirus genus) in the principal components analysis. (**g**) The percentage of samples positive for the three anellovirus genera in the study populations. (**h**) Percentage of samples that were positive for one, two or three clusters according to patient group.

A principal components analysis (PCA) on the aligned anellovirus sequences allowed the assessment of the anellovirus evolutionary space of the observed contigs and corresponding relationships to sequences in the reference database (Figure 6a). The plot clearly defines the boundaries of the three anellovirus genera known to infect humans, the *Alphatorquevirus*, *Betatorquevirus*, and *Gammatorquevirus*. Overall, all three genera were observed in both patient and control populations (Figure 6b–d). Sequences that were most often observed (>8 pools) belonged to the *Alphatorquevirus* genus (*n* = 10), followed by the *Betatrorquevirus* (*n* = 2) and *Gammatorquevirus* (*n* = 1). The *Alphatorque* genus was most frequently observed in two or more study groups compared to other genera (Figure 6f), although differences were not significant (Fisher’s exact test, *p* > 0.05). When comparing different study populations, *Alphatorqueviruses* were more frequently observed in both control and dialysis pools (94% and 92% of anellovirus positive samples, respectively), while viruses of the *Betatorquevirus* genus (90% of anellovirus positive samples) were more dominant in thalassemia pools (Figure 6g). Finally, in anellovirus-positive pools of both dialysis and thalassemia, the observed viral contigs covered all three clusters at a higher percentage, although these differences were not significant (Figure 6h, control: 47%, hemodialysis: 66% and thalassemia: 63%, Fisher’s exact test, *p* > 0.05). 

### 3.5. Pegivirus

**Figure 7:**
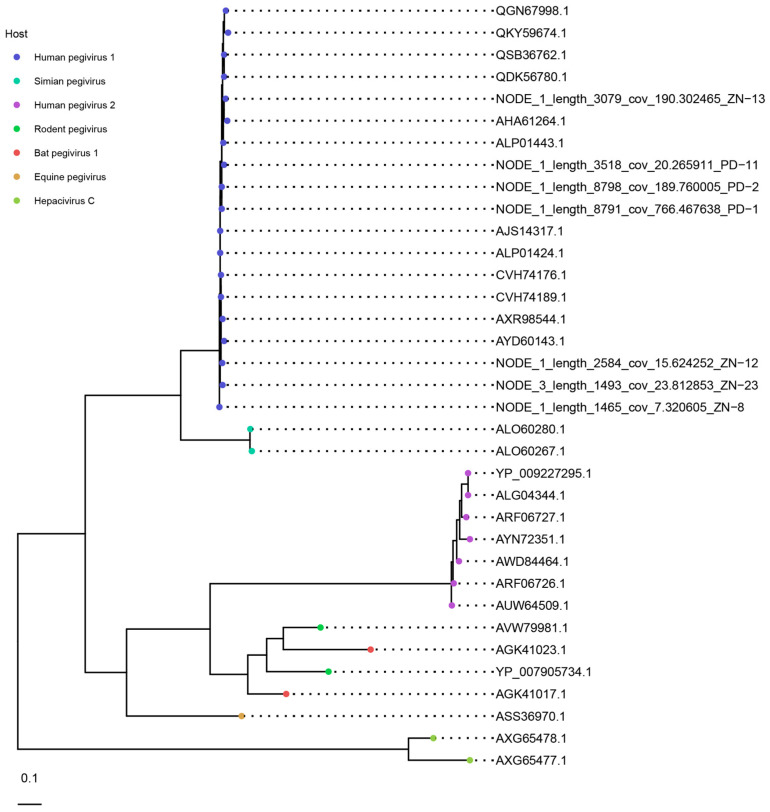
Maximum-likelihood tree of the predicted NS5B protein sequences (*n* = 7) from the pooled samples and NCBI reference sequences (*n* = 28).

In addition to the frequently found members of the *Anelloviridae* family, viruses belonging to the *Flaviviridae* were also observed in multiple pools. Remarkably, sequencing data demonstrated the presence of human pegivirus (HPgV) in pools of dialysis (6/32) and thalassemia (7/31) patients. None of the control pools were positive for HPgV. A total number of 104 HPgV contigs were assembled with an average size of 1040 bp (median 504 bp). We predicted ORFs from the annotated contigs and aligned protein sequences with NS5B (RNA-dependent RNA polymerase) reference sequences from human and non-human hosts to build a maximum likelihood tree. The phylogenetic analysis illustrates that the observed contigs cluster within the HPgV-1 clade (Figure 7).

## 4. Discussion

The blood virome remains an underrepresented fraction of the human microbiome in scientific literature. Little is known about the viruses that circulate in the blood stream of healthy and diseased individuals. In this study, we have characterized the blood virome of both healthy controls, thalassemia, and hemodialysis patients originating from Iran. Viruses detected in the blood of these multi-transfused patients are indicators of viruses that are circulating in the population and not covered by the screening panels used in blood banks [15,16]. Multiple viral families have been identified with the majority of samples harboring members of the *Anello*- and *Flaviviridae* families. In addition to these established members of the blood virome, other potentially clinically relevant viruses have been observed in samples of healthy donors (e.g., rotavirus and Merkel cell polyomavirus). An in-depth analysis of the *Anelloviridae* family revealed a diverse population of anelloviruses. Interestingly, members of the *Flaviviridae* (HPgV) were solely detected in patient samples.

A benchmark paper published in 2017 defined the human DNA blood virome in 8000 individuals and reported the presence of a wide variety of viral families [45]. These included known pathogenic viruses, e.g., hepatitis b/c virus and HIV, as well as non-pathogenic viruses of the *Anelloviridae* family. Here, we did not identify pathogenic viruses in control or patient pools that are covered by the blood screening panels used in blood banks (e.g., HBV/HCV and HIV). This confirmed our expectations since both donor and patient blood are screened on a regular basis. However, traces of Merkel cell polyomavirus, which does have pathogenic potential, were observed in both healthy blood donors and dialysis pools (Figure 2) [46,47]. Furthermore, segments of rotavirus A (*Reoviridae*), a common cause of gastroenteritis in children, [48] and adenovirus (type 41/F) have been observed in plasma pools of control patients. Even though most infections are asymptomatic infections, adenovirus can induce mild flu-like illness and gastroenteritis (subtypes 40 and 41/F) [49]. Recently, this virus has been associated with cases of idiopathic hepatitis in children, although, studies have not been conclusive so far [50,51]. In addition to adenovirus, some control pools were positive for adeno-associated virus (AAV, *Parvoviridae*). This virus requires a coinfection with adenovirus or herpesvirus for its replication and can remain latent in human leukocytes [52]. Reactivation might coincide with a reduced immunocompetence and the reactivation of herpesviruses [53]. Although prevalent in the blood of healthy individuals, AAV does not seem to be an immediate concern for blood transfusion safety.

We recovered both eukaryotic and prokaryotic viral genomes from the sequencing data. The analysis demonstrated the presence of bacteriophages with *Escherichia*, *Cronobacter*, *Citrobacter*, and *Corynebacterium* genera as their corresponding hosts (Supplementary Figure S6). Apart from the *Corynebacterium*, the observed prokaryotic virus contigs originated from hemodialysis pools. Individuals recruited in this study did not express clinical symptoms of infections. Therefore, the presence of phages and potential hosts could hint at asymptomatic infections and/or spill over from other body sites to the blood. For instance, ESRD has been associated with an impaired gut barrier function, which could facilitate the leakage of gut (microbiota) content into the blood circulation [54]. Additionally, nosocomial exposure during hemodialysis has been associated with an increased risk of bacterial infection [55]. Opportunistic infections could root from a reduced host-immunocompetence caused by underlying illness [56]. Finally, the presence of *Corynebacterium* phage in healthy blood donors could be related to skin microbiota contamination caused by blood donation (venipuncture).

The most widely observed viruses in the studied plasma samples belonged to the *Anelloviridae* family. This family consists of small single-stranded DNA viruses that are highly diverse and have a wide host range. Three genera have been identified in samples of human origin, including the *Alphatorquevirus*, *Betatorquevirus*, and *Gammatorquevirus* and were widely observed in the pooled samples. Remarkably, we observed an increasing trend in samples positive for all three genera in patient versus control pools (Figure 6). In current databases, the most detected species have been classified in the alphatorquevirus genus, similar to the dominant genus observed in the studied samples [57]. Our diversity analysis demonstrated the lack of anellovirus contigs sharing between different pools and study groups (Figure 5) (control vs. dialysis vs. thalassemia). This confirms previous findings that reported diverse inter-individual anellovirus communities characterized by a high personalized nature [58,59].

Anelloviruses were highly prevalent in healthy individuals and have previously been found in blood products prepared for transfusion [18,60]. Furthermore, evidence of transfusion-associated anellovirus transmission has been reported [58]. The transmitted anelloviruses could alter the recipient’s anellovirus community architecture. In our study, we did not observe traces of anellovirus similarities between the healthy donor population and patient groups. To our knowledge, the study population did not include known donor-recipient pairs. Furthermore, considering the highly personalized characteristics of the anellovirus community, we did not expect high inter-pool similarities for this group of viruses [59,61].

The patients that required hemodialysis received a higher number of blood products compared to thalassemia patients (Table 1). Interestingly, both the total eukaryotic abundance and relative abundance of viruses of the *Anelloviridae* family were higher in hemodialysis patients (Figure 3 and Figure 4, respectively). Previous studies have linked TTV (*Alphatorquevirus*) presence to blood transfusion frequency and time under treatment in hemodialysis patients [62]. Similar conclusions were drawn in Romanian individuals [63]. However, a study performed in Northern Iran applied a nested PCR method to compare the presence of TTV in both hemodialysis and thalassemia patients and observed no significant differences [64]. Inter-study comparisons can be affected by the used method to detect and quantify anelloviruses. Furthermore, in this study, the pooling of samples occurred according to the region of origin and did not consider transfusion information. Therefore, the potential impact of transfusion frequency could only be assessed on population level.

Although both groups of thalassemia and hemodialysis receive red blood cells, leucocyte reduction techniques are more frequently applied for blood products administered to thalassemic patients than hemodialysis cases in Iran. Even though we did not obtain specific data on the administered blood products, Iranian clinicians involved in this study indicated that leukocyte reduction was mostly applied to blood products prepared for thalassemia patients in this study. This intervention might significantly decrease the load of anelloviruses in administered red blood cells [65]. Longitudinal data that cover pre- and post-transfusion timepoints are needed to investigate this hypothesis.

Future studies should focus on the long-term dynamics of anelloviruses and their potential clinical use in hemodialysis patients. For instance, secondary infections significantly contribute to the increased morbidity and mortality in ESRD [66]. We have previously reported that a high anellovirus abundance could be associated with an increased risk of acquiring infections in liver transplant recipients [12]. Anelloviruses as potential immunocompetence biomarkers could be used to assess the individual risk of infection in ESRD patients and support the prescription of personalized treatment strategies.

Besides *Anelloviridae*, we observed the presence of HPgV (*Flaviviridae*) in multiple pools of both dialysis and beta-thalassemia patients. Remarkably, none of the control pools were positive for these viruses, which might suggest clearance of the infection in healthy blood donors. HPgV is a single-stranded RNA virus of the *Flaviviridae* family and is phylogenetically closely linked to hepatitis C virus. HPgV is considered a non-pathogenic virus and widespread among the healthy population, with an estimated prevalence of 4% in healthy blood donors and 5–13% of people with anti-E2 antibodies that indicate previous exposure [67]. The non-pathogenic characteristic and its endemicity have prevented the implementation of routine screening in blood transfusion safety guidelines. Rejection of positive blood donations would greatly reduce the number of eligible blood products for transfusion. Nevertheless, the absence of screening increases the risk of transmitting HPgV through blood transfusion, which might explain the increased prevalence in our studied patient population compared to healthy blood donors. In the present study, all the annotated pegivirus contigs were classified as HPgV-1, the most frequently observed pegivirus genotype in humans [68]. Studies have suggested a delayed progression to acquired immunodeficiency syndrome in an HPgV and HIV-coinfected individual [69,70]. Another study demonstrated a protective effect and improved HIV infection outcome after acquiring HPgV through blood transfusion [71]. These findings highlight the potential clinical relevance of commensal viruses in blood transfusion practice.

Non-pathogenic viruses, such as pegi- and anellovirus, can be used as a marker of exposure and risk of acquiring viral pathogens. A recent paper reported the accumulation of non-pathogenic viruses in people who inject drugs and associated the acquisition of these viruses with the risk of HCV infection [72]. In our study population, pegivirus was only observed in the transfusion-receiving population. Furthermore, an increased anellovirus contig richness was found in hemodialysis patients. Both viruses contributed to an increased eukaryotic virus load (in terms of relative abundance) in the patient population, although only in the hemodialysis group was this significantly increased. These findings might result from an increased exposure to both viral families through blood transfusion practice. However, the absence of pegivirus in control samples is an unexpected finding considering the prevalence of this virus in healthy individuals. This could be explained by the clearance of the virus in healthy individuals [73], which requires testing for pegivirus antibodies to confirm this hypothesis.

Currently, there is no gold standard available for studies of virome communities in biological samples, both in terms of laboratory practice and bioinformatic analysis. We employed a sequencing method without targeted probes, which might affect the sensitivity to viruses with a low viral load. Additionally, the pooling of samples could also impact the detection of viruses, although this strategy enabled us to include a large number of individuals. Lastly, we conducted a cross-sectional study of the virome, which does not provide information on long-term dynamics and stability of the blood virome in these patients. We would strongly recommend that future studies carefully consider these limitations.

## 5. Conclusions

This study provided a comprehensive analysis of the blood virome in multiple-transfused patients and healthy blood donors. We observed a high prevalence of viruses of the *Anelloviridae* family and pegiviruses, of which the latter were only detected in transfusion recipient groups. The observed anellovirus species diversity was not clearly associated with the underlying health conditions (patients versus healthy). Remarkably, an increased eukaryotic abundance was observed in the hemodialysis group, which was primarily driven by the high abundance of anelloviruses. This could possibly be associated with a higher blood transfusion frequency. In addition to anellovirus, we observed genome fragments of potentially pathogenic viruses, including rota-, adeno-, and polyomaviruses in healthy blood donors. Of note, current blood screening strategies do not include the surveillance for these viruses. Future studies are needed to investigate the clinical relevance of these viruses in the blood and blood transfusion context.

## Figures and Tables

**Table 1:** Demographic information related to multiple-blood-transfused patients and healthy blood donors.

Variable	Thalassemia (ZN)	Hemodialysis (PD)	Blood Donors (PCL)
Number of samples	155	149	100
Age (mean ± sd)	24.7 ± 8.5	45 ± 10.4 ^1^	41 ± 9.7 ^1^
Sex (male/female)	79/21	58/41	90/10
Transfusion/year (mean ± sd)	19 ± 6	92 ± 55 ^2^	NA
Transfusion range	6–40	24–264	NA
Transfused product	Red blood cell	Red blood cell	NA

NA: Not applicable, ^1^ Kruskal–Wallis test and post-hoc Dunn’s test *p* < 0.05, ^2^ Wilcoxon rank-sum test, *p* < 0.05.

## Data Availability

The data presented in this study are openly available in the NCBI’s Sequence Read Archive (SRA) database under the Bioproject with accession number PRJNA983534. The sequences datasets used for phylogenetic analysis are available at https://github.com/MarijnThijssen/Virome_Multiple_blood_transfusion/tree/main (accessed on 16 June 2023).

## Supplementary Materials

The following supporting information can be downloaded at: https://www.mdpi.com/article/10.3390/v15071425/s1, Figure S1. Viral (sub)families with high prevalence in the blood virome of the studied population. Figure S2. Viral (sub)families with medium prevalence in the blood virome of the studied population. Figure S3. Viral (sub)families with low prevalence in the blood virome of the studied population. Figure S4. Viral (sub)families with the lowest prevalence in the blood virome of the studied population. Figure S5. Length distribution of all (un)annotated contigs. Figure S6. Host prediction of the observed bacteriophage contigs. Figure S7. Length distribution of the anellovirus contigs. Table S1. Relative abundance of the observed eukaryotic viral families and prokaryotic viruses. Table S2. List of *Anelloviridae* accession numbers used to build the phylogenetic tree.
