# Supplementary material for: Characterization of the Human Blood Virome in Iranian Multiple Transfused Patients
Source: Viruses. 2023 Jun 23;15(7):1425. doi: 10.3390/v15071425 (PMC10386462; doi:10.3390/v15071425)
Supplement: Supplementary file 1 [file viruses-15-01425-s001.zip › Supplementary Figure S5.pdf]

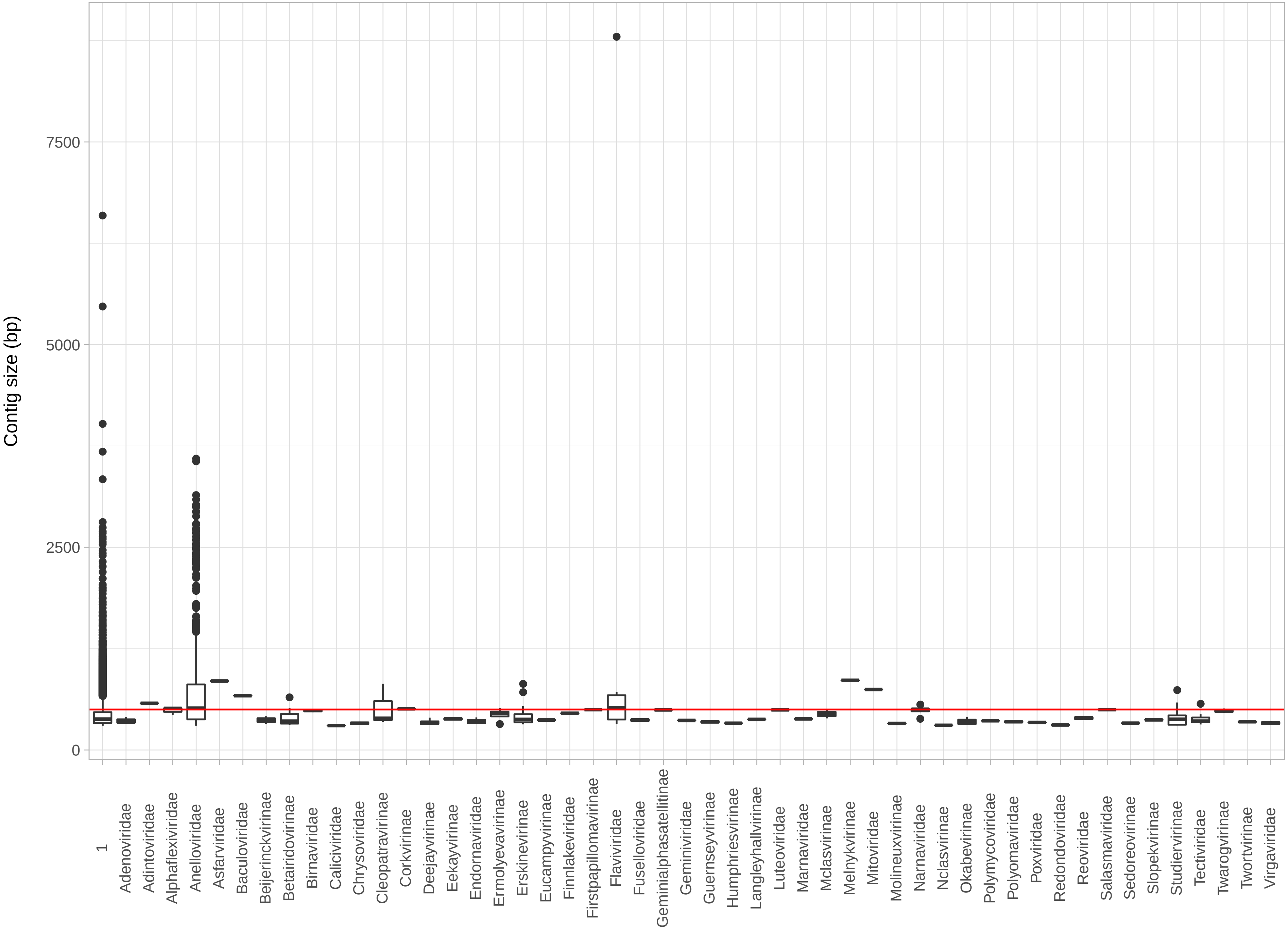

Contig size (bp)
7500
5000
2500
0
1
Adenoviridae
Adintoviridae
Alphaflexiviridae
Anelloviridae
Asfarviridae
Baculoviridae
Beijerinckvirinae
Betairidovirinae
Birnaviridae
Caliciviridae
Chrysoviridae
Cleopatravirinae
Corkvirinae
Deejayvirinae
Eekayvirinae
Endornaviridae
Ermolyevavirinae
Erskinevirinae
Eucampyvirinae
Finnlakeviridae
Firstpapillomavirinae
Flaviviridae
Fuselloviridae
Geminialphasatellitinae
Geminiviridae
Guernseyvirinae
Humphriesvirinae
Langleyhallvirinae
Luteoviridae
Marnaviridae
Mclasvirinae
Melnykvirinae
Mitoviridae
Molineuxvirinae
Narnaviridae
Nclasvirinae
Okabevirinae
Polymycoviridae
Polyomaviridae
Poxviridae
Redondoviridae
Reoviridae
Salasmaviridae
Sedoreovirinae
Slopekvirinae
Studiervirinae
Tectiviridae
Twarogvirinae
Twortvirinae
Virgaviridae
