# Supplementary material for: Characterization of the Human Blood Virome in Iranian Multiple Transfused Patients
Source: Viruses. 2023 Jun 23;15(7):1425. doi: 10.3390/v15071425 (PMC10386462; doi:10.3390/v15071425)
Supplement: Supplementary file 1 [file viruses-15-01425-s001.zip › Supplementary Figure S7.pdf]

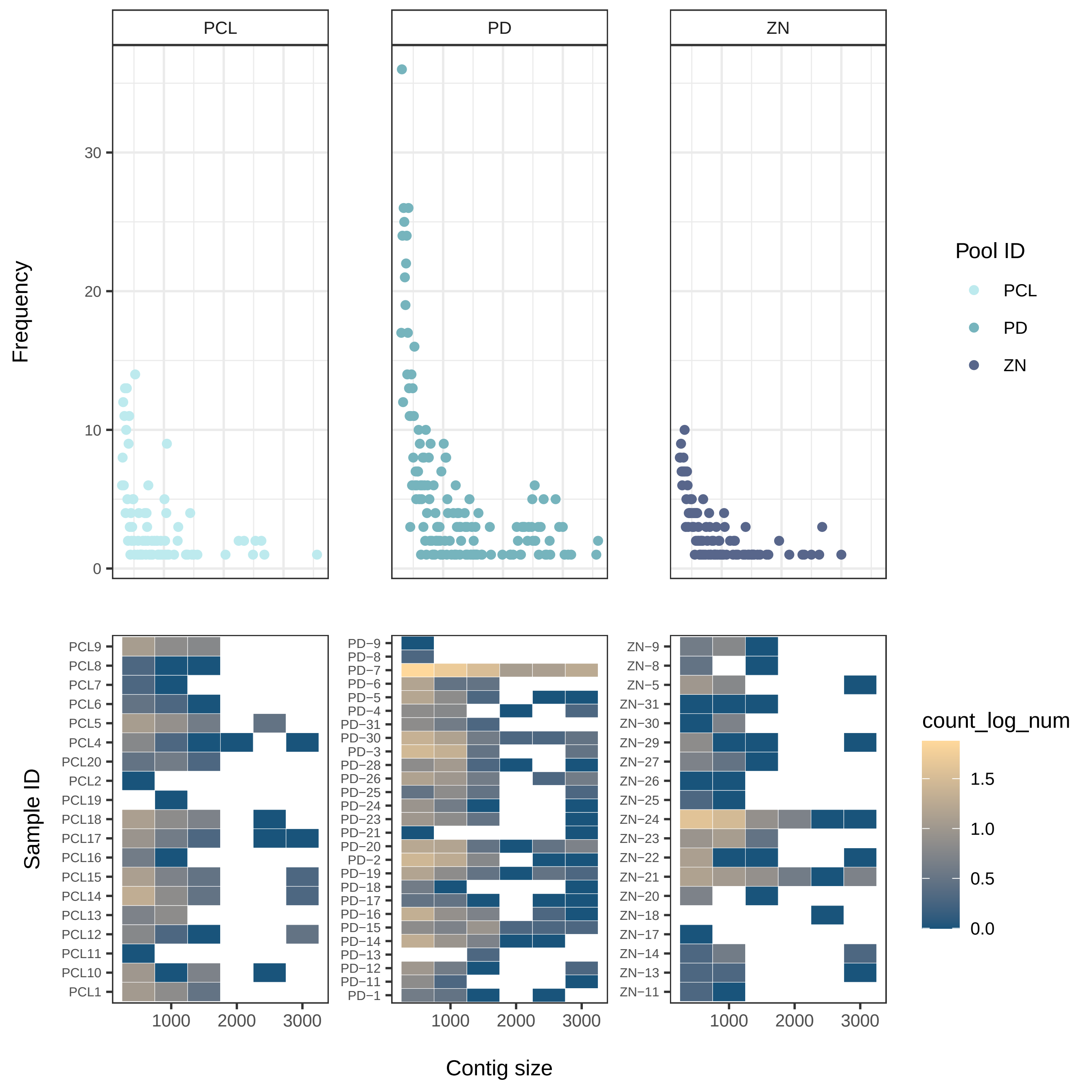

PCL
PD
ZN
Frequency
0
10
20
30
Pool ID
PCL
PD
ZN
Sample ID
PCL9
PCL8
PCL7
PCL6
PCL5
PCL4
PCL20
PCL2
PCL19
PCL18
PCL17
PCL16
PCL15
PCL14
PCL13
PCL12
PCL11
PCL10
PCL1
PD−9
PD−8
PD−7
PD−6
PD−5
PD−4
PD−31
PD−30
PD−3
PD−28
PD−26
PD−25
PD−24
PD−23
PD−21
PD−20
PD−2
PD−19
PD−18
PD−17
PD−16
PD−15
PD−14
PD−13
PD−12
PD−11
PD−1
ZN−9
ZN−8
ZN−5
ZN−31
ZN−30
ZN−29
ZN−27
ZN−26
ZN−25
ZN−24
ZN−23
ZN−22
ZN−21
ZN−20
ZN−18
ZN−17
ZN−14
ZN−13
ZN−11
count_log_num
0.0
0.5
1.0
1.5
1000
2000
3000
Contig size
