# Supplementary figures and images for: Characterization of the Human Blood Virome in Iranian Multiple Transfused Patients
Source: Viruses. 2023 Jun 23;15(7):1425. doi: 10.3390/v15071425 (PMC10386462; doi:10.3390/v15071425)

Animal Incl. Animal Other

Viral family

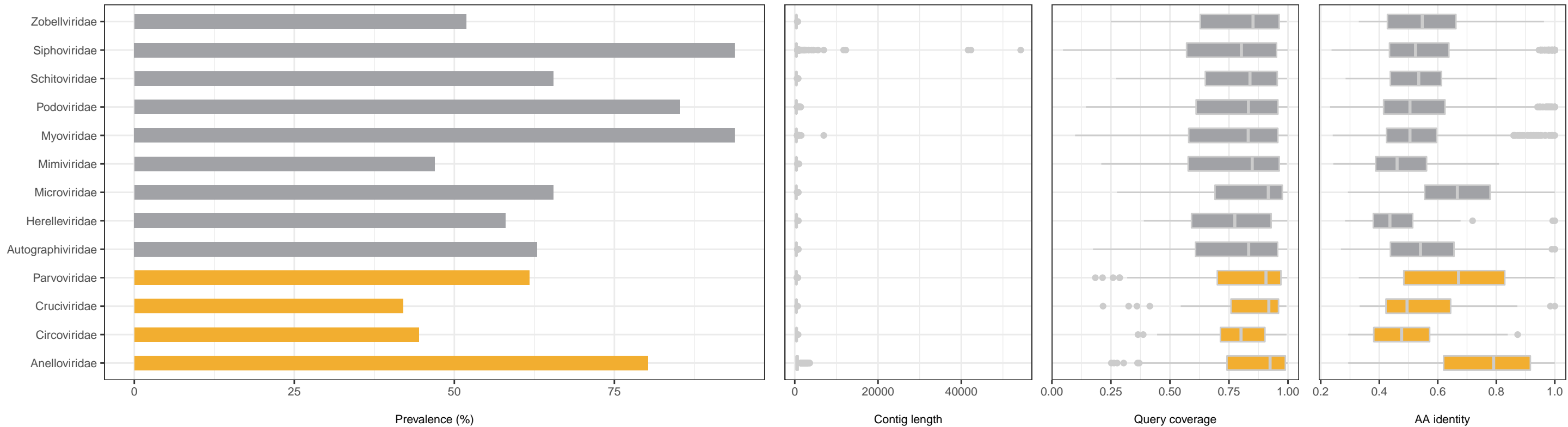

Supplement: Supplementary file 1 [file viruses-15-01425-s001.zip › Supplementary Figure S1.pdf]

Animal Incl. Animal Other

Viral family

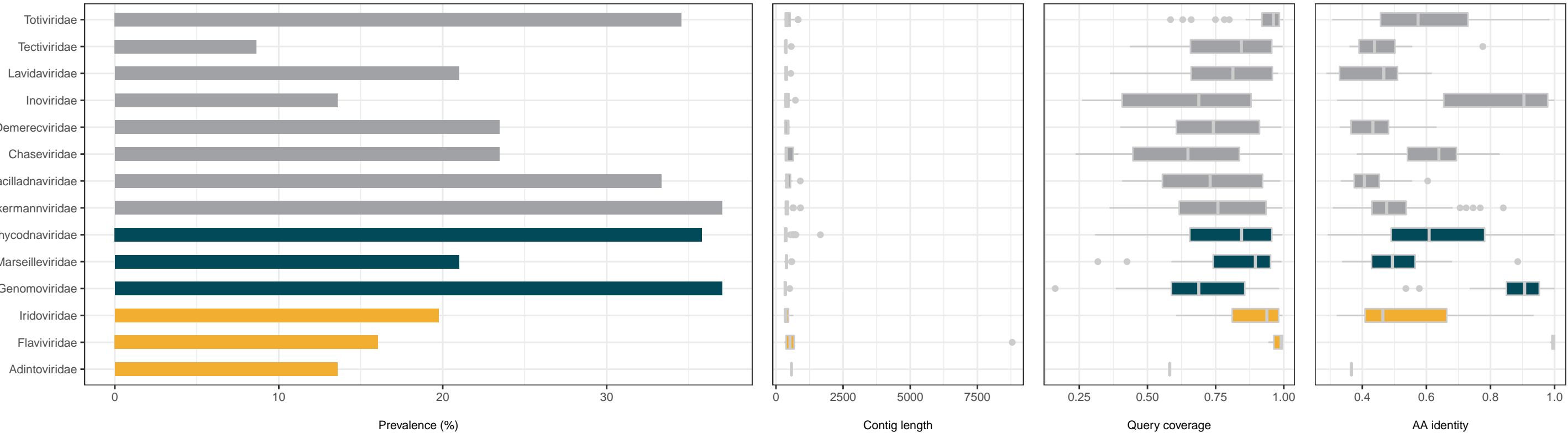

Supplement: Supplementary file 1 [file viruses-15-01425-s001.zip › Supplementary Figure S2.pdf]

Animal Incl. Animal Other

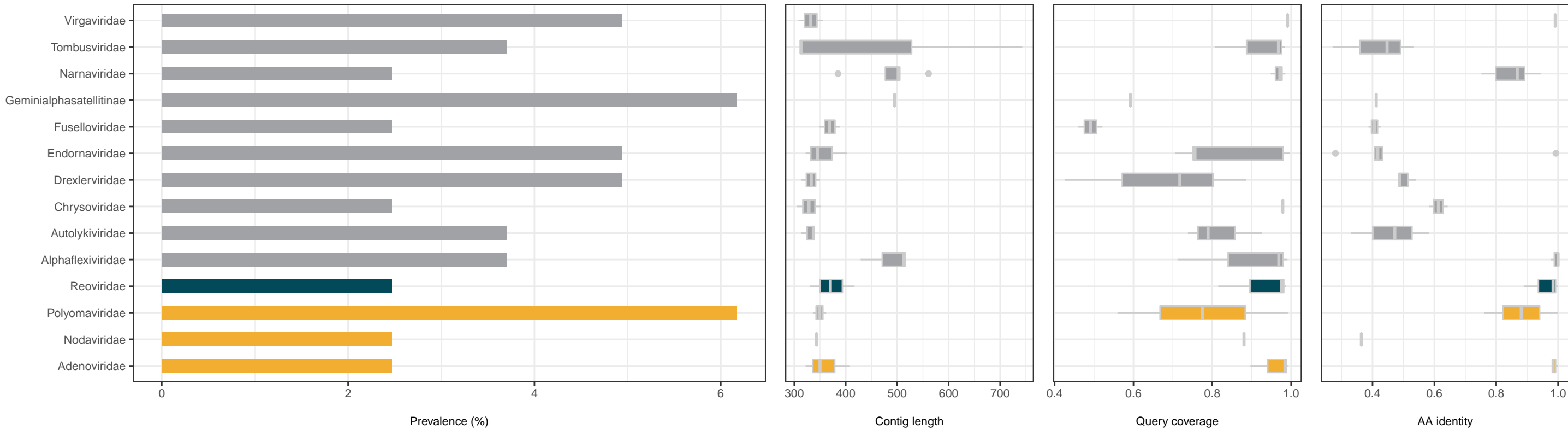

Supplement: Supplementary file 1 [file viruses-15-01425-s001.zip › Supplementary Figure S3.pdf]

Animal Incl. Animal Other

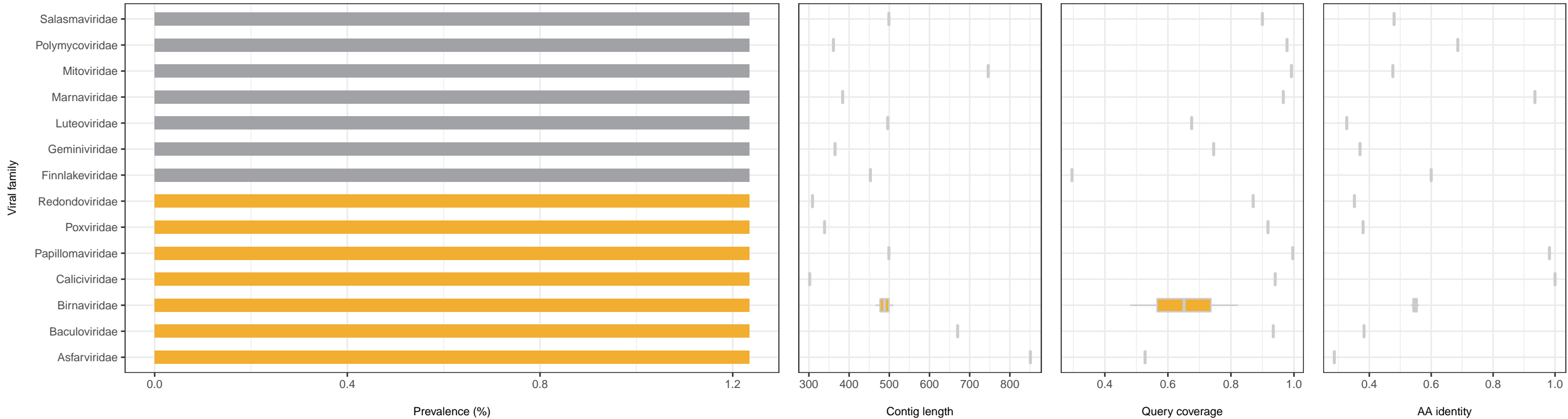

Supplement: Supplementary file 1 [file viruses-15-01425-s001.zip › Supplementary Figure S4.pdf]

**a**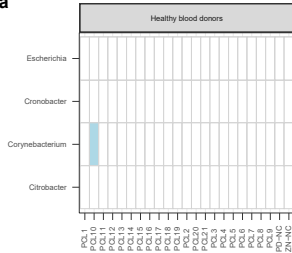**b**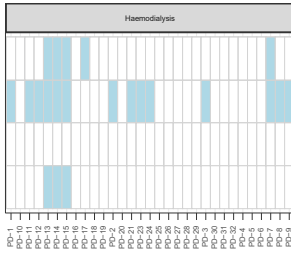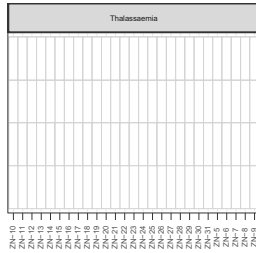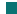

Environmental pathogen (Food, urinary tract...)

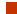

Skin and respiratory tract

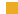

Gut

0

5

10

Supplement: Supplementary file 1 [file viruses-15-01425-s001.zip › Supplementary Figure S6.pdf]
